# The Outlines of General Pathology

**Published:** 1851-09

**Authors:** 


Art. VIII.—*The Outlines of General Pathology.* By M. L. Linton, M. D., Professor of the Theory and Practice of Medicine in the Medical Department of the St. Louis University. St. Louis: 1851. p.p. 205. 8vo.

We are glad to meet this unpretending little volume, with the contents of which we are already familiar, having read with great interest most of the essays which it embodies as they appeared, some years ago, in the *St. Louis Medical and Surgical Journal.* It professes to be simply the "outlines" of general pathology, but these outlines are sketched with a clearness and a force which induce a wish in the reader to see more from the pen of the author on the subject. They show him to be a philosopher, capable of eliminating and combining the truths of medical science. The principles unfolded in these pages are such as every student of medicine should make himself thoroughly master of, and we do not know a work on the subject which could be more advantageously placed in the hands of young students. The style is simple, clear and forcible, the matter well arranged, and the illustrations practical and adapted to the understanding of beginners. Prof. Linton's aim has evidently been to be useful.

The volume embraces twelve chapters, in which, among the topics discussed, are,—the causes of disease and their modus operandi, semiology; the action of remedies; general hyperemia and anemia; active congestion and inflammation; qualitative changes of the blood; hemorrhage, dropsy, and flux; pathological changes of the solids; Nosology; Medical doctrines.

It was Dr. Linton's intention, when he concluded to publish this volume, to enlarge his original essays; but he states in his preface that laborious professional duties have not left him the requisite leisure to carry out his design. We express the belief confidently, and take great pleasure in expressing it, that these pages contain the germ and elements of a work that, executed as the author is capable of doing, would be eminently creditable to him, useful, and enduring. Such an edition of it we hope soon to see. Y.

